# A Decrease in Branched-Chain Amino Acids after a Competitive Male Professional Volleyball Game—A Metabolomic-Based Approach

**DOI:** 10.3390/metabo14020115

**Published:** 2024-02-09

**Authors:** Taillan Martins Oliveira, Tathiany Jéssica Ferreira, Paula Albuquerque Penna Franca, Rudson Ribeiro da Cruz, Mauricio Gattás Bara-Filho, Fábio Luiz Candido Cahuê, Ana Paula Valente, Anna Paola Trindade Rocha Pierucci

**Affiliations:** 1 Laboratory of Food Development for Special Health Purpose and Education (DAFEE), Nutrition Institute Josué de Castro (INJC), Federal University of Rio de Janeiro, Rio de Janeiro 21941-902, Rio de Janeiro, Brazil; taillannut@ufrj.br (T.M.O.); tathianyferreira@ufrj.br (T.J.F.); pfranca@nutricao.ufrj.br (P.A.P.F.); rudsonribeiro@ufrj.br (R.R.d.C.); cahue@ufrj.br (F.L.C.C.); 2 Federal University of Juiz de Fora, Campus Universitario, Juiz de Fora 36036-900, Minas Gerais, Brazil; mauricio.bara@ufjf.br; 3 Medical Biochemistry Institute, National Center for Nuclear Magnetic Resonance, Universidade Federal Do Rio de Janeiro, Rio de Janeiro 21941-902, Rio de Janeiro, Brazil; anapval@bioqmed.ufrj.br

**Keywords:** nuclear magnetic resonance, exercise, biomarkers, metabolomics, recovery

## Abstract

A competitive volleyball game is a highly metabolic and physically demanding event for professional players. This study aimed to investigate whether a single game at the end of a preseason promotes changes in the biochemical markers of physical exercise responses and the metabolomic profile of professional volleyball players. This cross-sectional study included 13 male Brazilian professional volleyball players. Food intake, body composition, heart rate, physical movement variables, and blood biochemical indicators were evaluated. For non-target metabolomic analysis, serum samples were subjected to 500 MHz Nuclear Magnetic Resonance. Data analysis showed no significant difference in the biochemical indicators after the game (*p* > 0.05). The level of metabolites present in the groups of the main components (β-hydroxybutyrate, arginine/lysine, isoleucine, leucine, and valine) had decreased after the game. However, formic acid and histidine levels increased. Among the compounds not part of the main components, hypoxanthine and tyrosine increased, whereas low-density lipoprotein and very low-density lipoprotein levels decreased. After the game, the metabolomic profiles of players showed significant negative variations in essential amino acids (leucine, valine, and isoleucine). These decreases might be influenced by athlete diet and reduced glycogen storage due to lower carbohydrate intake, potentially impacting serum-essential amino acid levels via oxidation in skeletal muscle. The study provides insights for developing metabolic compensation strategies in athletes.

## 1. Introduction

Volleyball is a team sport with technical and tactical requirements for speed and strength [1]. As an anaerobic and aerobic sport, its metabolic demands are met primarily through phosphagen energy processes [2]. The game is characterized by multi-directional demands and numerous high-intensity efforts with short recovery periods, including acceleration, deceleration, jumping, frequent changes of direction, and actions with the ball [3,4]. In addition, volleyball has many requirements that combine physical and technical skills that determine most of the game’s elements, including attacking, serving, blocking, and setting [5].

The training load period is essential for planning the competitive season [6]. The pre-season period is when physical and muscular preparation is prioritized for sporting implications during the season [7,8,9]. After this phase, during the season, training will mainly consist of activities aimed at the player’s technical and tactical conditions [9]. Team sports may maintain technical and tactical training sessions with higher volume and intensity than physical preparations to sustain high performance and prevent injuries during the season [10].

Energy and substrate demands are high during training and matches, and moderate during training in the competitive season [11]. Adequate nutrient intake is critical for improving athletic performance, conditioning, recovering from fatigue after exercise, and avoiding injuries in volleyball [12]. However, players’ food intake may not meet the specific nutritional recommendations adjusted for physical training. Sesbreno et al. [13] found that elite male volleyball players consumed less energy than recommended for their sport. Pons et al. [14] observed reduced carbohydrate intake and increased protein and fat intake, as well as micronutrient inadequacy. It was pointed out that players had an impaired ability to adapt to and recover from training during a critical segment of the competitive season. Failure to meet the recommended nutritional needs may affect players’ metabolic and physiological capacity for exercise [15].

Evaluating recovery, inflammation, and muscle damage biomarkers is essential for assessing players’ performance and recovery. Thus, the aim of this study is to better comprehend the physiological, dynamic, and integrative aspects of nutrition and sports training [16]. Previous studies have reported increased creatine kinase (CK) levels after the preparatory period, suggesting a catabolic state and increased muscle damage in elite volleyball players [17,18]. In addition, assessing the non-targeted metabolite profile may provide a more comprehensive understanding of the overall metabolic state of players [9].

Metabolomics is a promising tool for studying the physiology and metabolism associated with exercise [19,20]. A recent review highlighted the impact of metabolic changes during exercise [20]. However, few studies have focused on volleyball players. Zhou et al. [21] obtained serum and urine metabolite markers in female adolescent volleyball players within 2 weeks of strength–endurance training using a metabolomic approach and biochemical marker analysis. The altered metabolites were mainly involved in energy, lipid, and amino acid metabolism, and female players had a greater propensity to oxidize lipids as their primary energy source [21].

Furthermore, exercise-induced oxidative stress occurred, as shown by the reduced glutathione and increased blood malondialdehyde and oxidized glutathione [21]. Metabolomics can reveal global metabolic perturbations and identify biomarkers associated with exercise-related disorders during crucial training periods for the preparation of players [21]. Therefore, this approach may also provide information relevant to analyzing the acute effects of a competitive volleyball game and its impact on players’ nutrition and recovery.

Consequently, this study aimed to investigate whether a single game at the end of the preseason can promote changes in the biochemical markers of physical exercise responses and the metabolomic profile of professional players. The present study hypothesized that acute stimulation represented by a game at the end of the preseason could change metabolite concentration relevant to future nutritional interventions aimed at players’ rapid recovery.

## 2. Materials and Methods

### 2.1. Study Design and Participants

**Figure 1:**
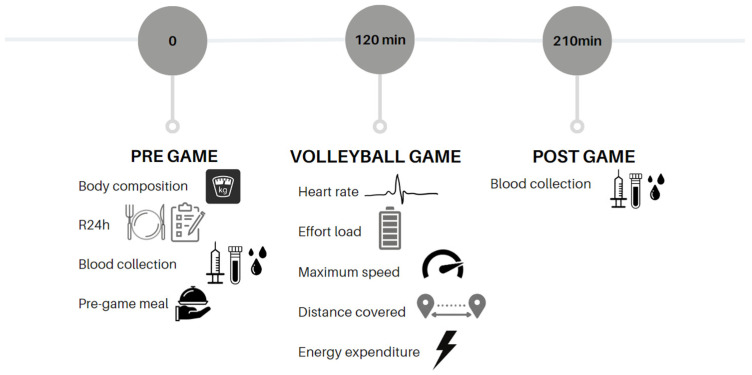
Study design.

This cross-sectional study included young Brazilian professional volleyball athletes. The experiment was conducted on the day of a regional championship game in Rio de Janeiro, Brazil. The analyses were carried out in the nutritional assessment laboratory (LANUTRI) of the Federal University of Rio de Janeiro (UFRJ). Initially, the players underwent a 24 h diet recall and electrical bioimpedance body composition analysis, followed by blood sample collection analyses that lasted 20 min. They then had a pre-game meal of their own choice (non-specific) 2 h before the game. The game lasted for 1 h and 30 min. Immediately after the match, another blood sample was collected (Figure 1). Notably, the researchers did not influence the pre-game meal, which is consistent with a “real-life” scenario.

The study sample comprised 13 men with a mean age of 21 years (±3.54), all post-pubescent, from a professional volleyball club in Juiz de Fora, MG, Brazil. The inclusion criterion was being an athlete registered by the club for the competition. Volunteers who met the inclusion criterion and agreed to participate in the study signed a free and informed consent form. The exclusion criteria were non-attendance at the evaluation, non-adherence to the protocol necessary to perform the evaluations, consumption of supplements that mask the test results, recovery from a muscle injury, or deviation from the training routine.

The study was conducted in accordance with the Declaration of Helsinki, and ethical clearance was obtained from the University Hospital of the Federal University of Rio de Janeiro (protocol number 58179716.3.0000.5257).

### 2.2. Food Intake

Food intake was assessed using a 24 h recall (R24h) regarding energy content (kcal), macronutrients (carbohydrates, proteins, and lipids), and micronutrients (calcium, iron, zinc, phosphorus, selenium, copper, magnesium, manganese, potassium, sodium, B vitamins (B1, B2, B3, B6, B9, and B12), and other vitamins (A, C, D, and E)). The assessment of food intake for all participants was carried out by a trained nutritionist.

The quantitative analysis of the energy and nutrients ingested was performed by converting the household measurements reported by the athletes into weight/volume measurements using the Table for Assessment of Food Consumption in Home Measures [22]. Nutritional composition was evaluated according to the United States Department of Agriculture (USDA) [23] table. The American Dietetic Association (ADA) recommendations (2016) [15] were considered to verify the adequacy of macronutrient intake in athletes. Dietary Reference Intakes (DRI) [24,25] were used to assess the adequacy of the intake of the analyzed micronutrients.

### 2.3. Anthropometric and Body Composition Evaluation

The assessment of anthropometric and body composition for all participants was carried out by a trained nutritionist. A Filizola^®^ balance scale, with a 0.05 kg scale, was used to assess body mass. A stadiometer, Altura Exata^®^, consisting of a metric scale with a resolution of 1 mm, was used to measure the height of the individual standing barefoot [26]. The athletes underwent bioimpedance testing using Byodinamics^®^ 450 equipment, a multifrequency direct bioimpedance analyzer using a bipolar system with four tactile electrode points at two anatomical points (right hand and right foot). Participants were instructed not to drink alcohol or caffeine, to avoid exercise, to discontinue the use of diuretics, and to fast for 4 h before the examination. The athletes were advised to wear only underwear. Each athlete was placed supine and asked to remove metal ornaments; their lower limbs were separated, the upper limbs were placed away from the trunk, and the electrodes were attached to the hand and feet. After the tests, fat-free mass, fat mass in kilograms (kg), and percentages were obtained.

### 2.4. Monitoring of Physiological Variables during the Game

During the match, variations in the athletes’ heart rate, effort load, maximum speed, distance covered, and energy expenditure were evaluated using an evaluation system (Polar Team System Pro version 1.4^®^). The equipment included a global positioning system tracker based on the athlete’s movement on the court and data from cardiometers. The data was subsequently processed by the equipment system and made available. The athletes received and installed the equipment on their backs before the game.

### 2.5. Blood Collection

Blood samples were collected before the game at LANUTRI, and immediately after the volleyball game in the game gym, by the same trained nurse. A puncture was made through the antecubital vein using a disposable material, and blood was collected in vacuum tubes. The blood was collected in specific tubes for serum and plasma, centrifuged at 1500× *g* for 15 min at 4 °C, aliquoted, and stored in a freezer at −80 °C until analysis [27].

### 2.6. Biochemical Analysis

Uric acid (UA) (UR3825), alanine aminotransferase (ALT) (AL3801), aspartate aminotransferase (AST) (AS3804), creatine kinase (CK) (CK3812), triglycerides (TR3823), lactate (LC3980), cholesterol (CH3810), glucose (GL3815), low-density lipoproteins (LDL) (CH3841), high-density lipoproteins (HDL) (CH3811), hydroxybutyrate (RB1007) and total antioxidant capacity (NX2332) in plasma were analyzed at Laboratorio de desenvolvimento de alimentos para fins especiais e educacionais (Lab DAFEE), UFRJ. Notably, all biochemical analyses were performed following the manufacturer’s instructions using commercial kits (Randox^®^ Laboratories-US Ltd., Kearneysville, WV, USA and a semiautomatic biochemical analyzer (Daytona RX, Randox^®^ Laboratories-US Ltd., Kearneysville, WV, USA). All measurements were performed in triplicates.

### 2.7. NMR Spectroscopy

A serum aliquot from each blood sample was used for metabolomic analysis using nuclear magnetic resonance (NMR) spectroscopy at Centro Nacional de Ressonância Magnética Nuclear Jiri Jonas (CNRMN), UFRJ. Samples were prepared by mixing 400 µL of serum with 200 µL of phosphate buffer (pH 7.4 with 10% deuterated water (D2O) and 0.5 mM sodium azide). All NMR experiments were performed as described by Oliveira et al. [28] using a 500 MHz Avance DRX spectrometer (Bruker Biospin, Karlsruhe, Germany) at 298 K. One-dimensional 1H NMR spectra were obtained using a standard 1 D Carr-Purcell-Meiboom-Gill pulse sequence to suppress signals from macromolecules through a T2 filter (84 ms) using 1024 scans. Notably, all spectra were referenced to the chemical shift of the anomeric proton signal of a-glucose at d 5.22 ppm, and the edge effects were evaluated by overlaying all spectra using TopSpin 3.2 (Bruker Biospin, Rheinstetten, Germany). Two-dimensional 1H–1H TOCSY spectra were acquired with acquisition parameters of 4096 × 512 points for selected samples to confirm metabolite assignment. The spectral width was 12,934 Hz, with a relaxation delay of 3 s and a spin-lock time of 60 ms. The assignment of resonances for the whole spectrum was performed following the literature [29], using the Human Metabolome Database (http://www.hmdb.ca/ accessed on 30 September 2022) [30]; an in-house database of one- and two-dimensional NMR spectra of reference compounds and two-dimensional TOCSY experiments were used for the purposes of confirmation.

### 2.8. Statistical Analysis

The number of participants in the study was determined using convenience sampling, given the impossibility of selecting samples by other means and the difficulty of obtaining volleyball players as volunteers for experimental procedures.

Data normality from all analyses performed in this study was tested using the Shapiro–Wilk test. Data were expressed as mean (±standard deviation), including a 95% confidence interval. A paired *t*-test was performed, with *p* < 0.05, to compare biochemical markers at the two time points. Spearman’s correlation was used to test the correlation between the data. Statistical analyses were performed using GraphPad prism^®^ version 8.0 software.

A bucket table generated from the NMR spectra acquired from blood serum samples was used to analyze the metabolomic data. Comparisons between time points were performed using a paired *t*-test. The variation trend was verified by an Empirical Bayes Analysis of a Microarray (EBAM). EBAM is a biotechnological tool based on a posterior probability of effects for each metabolite with a minimum number of a assumptions [31].

Principal component analysis (PCA) was performed to verify the degree of influence the group of identified metabolites had on the changes between the moments. Pearson’s and Spearman’s correlations were used to evaluate the correlations between the identified metabolites. All analyses were performed using MetaboAnalyst 5.0.

## 3. Results

Table 1 shows the anthropometric and body composition variables of the volleyball players who participated in this study.

The estimated nutrient intake of volleyball athletes is shown in Table 2. Protein intake (g/kg) was within the current recommendations for athletes; however, carbohydrate intake was below, whereas lipid intake was higher. Micronutrient intake was considered adequate based on the daily recommendations for the general population.

During the game, the athlete’s maximum heart rate was 198 bpm (±18.86), the total distance covered was 4620 m (±969.92), the maximum speed reached was 25 km/h (±3.40), the training load score was 224 (±83.20), and the estimated energy expenditure was 1856 kcal (±375.01).

The two moments analyzed showed no statistical differences in the biochemical biomarkers before and after one volleyball game (*p* > 0.05). The analyzed blood markers Alt, AST, glucose, UA, triglycerides, LDL, and HDL were within the recommended ranges (Supplementary Table S1).

**Figure 2:**
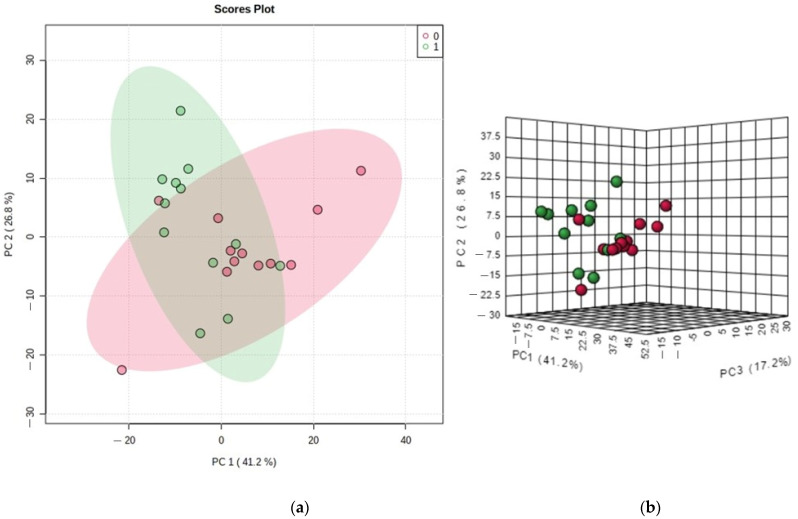
Principal component analysis (PCA) of the behavior of the main components and the difference in the pre-and post-game moments. (**a**) Changes in the metabolite profiles of athletes; (**b**) the main components (PC) 1, 2, and 3 and variation in the metabolite profiles between the selected moments before (Time 0: red) and after the game (Time 1: green).

In metabolomic analysis, the spectra showed 125 peaks, resulting in the identification of 66 different metabolites in the human metabolite database. Principal component analysis (PCA) performed on the blood NMR metabolome, shown in Figure 2, revealed that the volleyball game induced significant changes in the athlete’s metabolite profile (Figure 2a). The score plot g (Figure 2b) demonstrated that the main components (PC) 1, 2, and 3 explained 84.7% of the variation in the metabolite profile between the analyzed moments. There were three components from PCA, which were composed of the identified metabolites (and their respective loadings, which explains the impact of each metabolite on its respective component) as follows: PC 1: isoleucine (0.57), leucine (0.50), valine (0.45), arginine/lysine (0.09), formic acid (−0.08), and histidine (−0.03); PC 2: carnitine (−0.67), glutamate (−0.51), acetate and acetone (−0.40), acetyls (−0.24), and trimethylamine (0.20); PC 3: sarcosine (0.68), albumin (0.43), citrate (0.48), taurine (0.20), 3-hydroxybutyrate (0.17), and LDL/very low-density lipoprotein (VLDL) (0.08).

**Figure 3:**
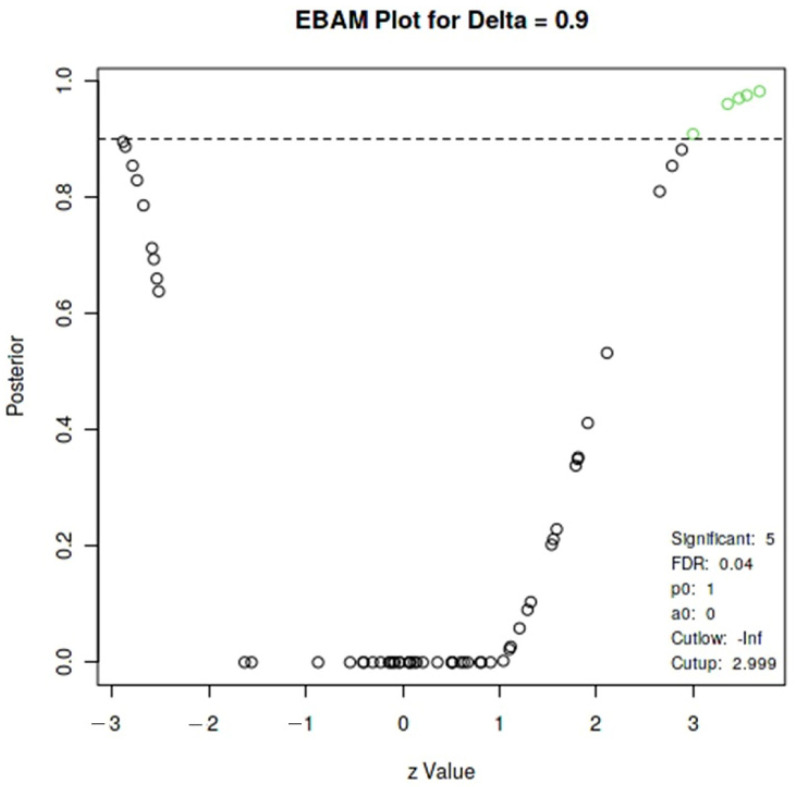
Empirical Bayes Analysis of a Microarray (EBAM) of the variation of the metabolites found in the study. False discovery rate (FDR). aO = 90th percentile.

A comparison between metabolites before and after treatment is shown in Table 3, and a metabolic change profile is shown in Figure 3. The levels of metabolites presented in PC1, β-hydroxybutyrate, arginine/lysine, isoleucine, leucine, and valine had decreased after the game. However, formic acid and histidine levels increased. Among the compounds that were not within any of the three main components from PCA, hypoxanthine and tyrosine increased, whereas LDL/VLDL decreased. In the comparative analysis of metabolite profiles, no differences were observed in the behavior of metabolites before and after the match between the starters and reserves (*p* > 0.05).

The EBAM analysis in Figure 3 presents a trend model for metabolite variation considering each metabolite’s expected variation. In the subsequent period, this analysis finds a false discovery rate of >0.04 and an a posteriori variation >0.90 as the expected behavior. It was observed that the variation in only four metabolites occurred significantly, as their posterior probability was ≥0.9, all being amino acids: isoleucine (posterior = 0.98, false discovery rate (FDR) = 0.01), leucine (posterior = 0.97, FDR = 0.02), valine (posterior = 0.97, FDR = 0.02), and Isoleucine.1 (posterior = 0.96, FDR = 0.03). The green point near the dotted line in the graph was discarded (Valine.1, posterior = 0.90, FDR = 0.09). Other variations were not significant in the analyzed samples. No correlations existed between metabolites and body composition variables, food intake, physiological variables during the game, or biochemical markers (*p* > 0.05).

## 4. Discussion

This study demonstrates the metabolic changes in male professional volleyball players induced by a competitive game using proton nuclear magnetic resonance metabolomics. Athletes were at the end of a training microcycle during pre-season preparation. Our main results showed that volleyball games induced significant changes in metabolites, which followed different patterns of variation. The metabolites 3-hydroxybutyrate, arginine/lysine, isoleucine, leucine, valine, and LDL/VLDL showed negative variations. However, the levels of formic acid, folate, histidine, hypoxanthine, and tyrosine showed positive variations. There was no correlation between the metabolite profile and the anthropometric, dietary, and biochemical evaluation variables. No significant differences were found in the biochemical variables analyzed, indicating that the athletes probably adapted to the physical effort experienced during the game.

The anthropometric and body composition characteristics of athletes in this study align with what is reported in the literature among male athletes. León-Guereño et al. [32], in their study of male athletes, reported body weight (80.4 ± 6.3), BMI (24.1 ± 1.3), and body fat percentage (8.0 ± 1.2). Fields et al. [33] evaluated athletes in an age range (18–24 years) close to that analyzed in the present research and showed that body composition parameters were lower than the present study for fat-free mass (72.5 ± 4.6) and for fat mass (13.6 ± 6.5) in absolute weight. However, when the results were compared in percentage values, the athletes in this study had lower fat mass. Despite the similarity of results, differences observed for BF%, FM, FFM, and BM are likely attributed to the physiological demands associated with specific sports and positions therein.

Assessing dietary intake helps to identify adherence to sports nutrition recommendations and makes it possible to intervene nutritionally to optimize performance. Protein intake was based on the recommendations for athletes; however, carbohydrate and lipid intakes were below and above the recommendations, respectively [15]. The low consumption of carbohydrates, below the recommended level, can limit performance during exercise and adequate athlete recovery [34], thus demanding supplementation for this nutrient from carbon skeletons. Continuously exacerbated lipid consumption can induce increased body fat stores, thereby increasing fat mass, which can harm performance [11]. Carbohydrates are the primary energy sources during intense and intermittent sports, such as volleyball, and are essential for maintaining optimal blood glucose levels for muscle contraction and recovery [11]. Low carbohydrate availability during exercise can lead to gluconeogenesis from non-carbohydrate substrates such as amino acids and ketone bodies [35]. Blood glucose levels were maintained during the game, indicating that the athletes’ energy metabolism maintained the availability of substrates for exercise. However, to do so, amino acids may be mobilized for gluconeogenesis. Amino acids are compounds present in the structural formation of the musculature and act as signals for post-exercise muscle recovery stimuli, so they are essential for athlete recovery [36]. Therefore, these compounds should not be mobilized to produce energy during exercise.

The main changes observed in the metabolites after the game were in amino acids and their derivatives. The decreased levels of leucine, valine, and isoleucine could be associated with this study’s low carbohydrate consumption profile. Blomstrand et al. [37] reported a decrease in branched-chain amino acid (BCAA) concentrations in the plasma of endurance-trained men with low glycogen storage induced by 60 min of cycling. This decrease was also observed in skeletal muscle when glycogen storage was low [38].

The direction of BCAA (protein synthesis signaling or oxidation) depends on the energy demand and storage. Since the athletes in this study demonstrated a low carbohydrate consumption profile, and the game lasted approximately 100 min, the decrease in BCAA concentration may be due to the high energy demand and low muscle glycogen storage. Therefore, it is only possible to ensure a cascade of protein synthesis for muscle recovery if the amino acids used for energy production are replaced by endogenous metabolism or dietary intake [15]. Replacement is important because the BCAA can act as a signal to the mammalian target of the rapamycin signaling pathway (*mTOR*) and its cascade targets, such as the phosphorylated 70s6 kinase p70s6k and eukaryotic initiation factor 4e-binding protein 4e-bp1, which modulate protein synthesis and muscle growth, and over time may result in hypertrophy [36].

There are many pathways through which amino acids are involved during exercise; however, these compounds could even be used as energy sources [39]. This study’s variations in amino acids were consistent with those described by Pechlivanis et al. [40], who found an increase in histidine and a decrease in leucine, valine, isoleucine, and arginine/lysine in 14 moderately trained healthy men who participated in two training programs that lasted 8 weeks and involved a series of maximal runs of 80 m separated by 10 s or 1 min of rest. This was consistent with the findings of Berton et al. [41]. Ten young, healthy men performed four series of 10 repetitions at 70% intensity, ending with a maximum repetition in the leg press and knee extension exercises, followed by a time course of up to 60 min, with a decrease in leucine, valine, and isoleucine. The variation in BCAAs detected using NMR analysis has been described in the literature; however, this variation may not be presented as a pattern. Amino acid levels can vary positively or negatively depending on the type of sport, exercise load, and intensity [35,42].

Other amino acids can promote exercise performance acting as regulators of energy production. Carnosine can buffer pH decrease during high-intensity exercise and, thus, promote performance [43]. In serum and tissues, Carnosinase-2 can degrade carnosine into β-alanine and L-histidine in blood and its activity determines the content of carnosine. After carnosine degradation, β-alanine and L-histidine can be transported into skeletal muscle cells [44]. An increase in histidine was found in this study, possibly suggesting, an increase in carnosine degradation in the blood.

For lipid metabolism, the combination of significant variations found in this study is consistent with the variation found after an exercise session that used strength and prolonged duration, such as in a volleyball game, which results in alterations in lipid metabolism, oxidative stress, and amino acid metabolism [45]. We observed higher HDL and lower LDL and triglyceride levels in the participants after the game. The exercise was associated with an increase in HDL cholesterol and a decrease in LDL cholesterol and triglycerides. Exercise, apart from inducing quantitative alterations in serum lipids, exerts a beneficial impact on HDL`s particle maturation, composition, and functionality. Acute or chronic prolonged endurance exercise performed at low to moderate intensity increases HDL levels as a result of enhanced lipid/lipoprotein metabolic activity. Further, the impact on HDL appears to be more pronounced when aerobic exercise rises during continuous effort [46].

We identified a significant variation in levels of hydroxybutyrate, a metabolite whose function is associated with lipid metabolism, as an intermediary in the production of ketone bodies, compounds that can be used for energy production [47]. Our results are consistent with those of a study of rowers who underwent a standardized 2-week training program and subsequently underwent field tests [45].

These results may be associated with a decrease in glycosidic compounds and glycogen reserves or impairment in carbohydrate oxidation. This information is vital for food and nutrition science, as it highlights the need to observe whether this deficiency results from inadequate consumption of macronutrients, mainly carbohydrates. This is consistent with our study, as the consumption of macronutrients was below the recommended level for carbohydrates but higher for lipids and proteins. Comparing the data found with the findings in the literature involving professional volleyball teams in Brazil, it was found that in both cases, only protein consumption occurred within the recommended standards [48]. These findings are consistent with those of another study on male athletes who showed the same behavior regarding macronutrient consumption [13]. Finally, the profile of overall low diet quality among athletes has been consistently reported [14].

Hypoxanthine is a predictor of physical capacity in professional athletes. Lower post-exercise hypoxanthine levels are associated with better sports performance [49]. Statistical analyses of the relationship and association between the study performance variables and metabolites and biomarkers were carried out, but no significant results were found. This performance improvement is explained by the lower concentration that leads to the inhibition of Hx-guanine phosphoribosyl transferase, an enzyme involved in the production cascade of purines and nitrogenous bases that constructs nucleotides, reducing their flow in skeletal muscles, an adaptation that is mediated through training [50].

The limitations of this study are the relatively small sample size, the inability to assess body composition using a reference method such as a dual energy X-ray absorptiometry, and the use of only GPS to monitor the game. The assessment of dietary intake, the evaluation of athletes during competitive matches, and similar metabolomic and physiological profiles among athletes are some strengths of the present study. Further, investigating the metabolomic profile of professional players after a single game at the end of the preseason can provide valuable data to enhance performance, optimize training, and improve the overall well-being of athletes in professional sports. Future studies should have larger sample sizes and verify the long-term effects of repeated games throughout the season, or the impact of cumulative fatigue of volleyball matches on the modulation of biochemical and metabolomic profiles.

Practical applications: Based on this study’s results, special attention should be paid to carbohydrate consumption. Evidence shows the positive effect of low carbohydrate consumption during the preseason [51]. Indeed, a slight increase in glucose intake may prevent a decrease in BCAA in plasma, contributing to muscle recovery after a match. Furthermore, athletes should have diets oriented to avoiding the metabolic mobilization of amino acids for energy production pathways. As food consumption was assessed by a single 24 h dietary record, we cannot state that it reflects the athletes’ habitual food consumption. However, it was possible to highlight the need for nutritional guidance for athletes.

## 5. Conclusions

The metabolic and biochemical profile of male athletes before and after a regional championship volleyball game showed significant negative variations in essential amino acids (leucine, valine, and isoleucine) and positive variations for formic acid, folate, histidine, hypoxanthine, and tyrosine. Our results suggest that, although plasma BCAA normally decreases after intense physical exercise, this variation can be potentialized by the athletes’ diet, as lower glycogen storage due to low carbohydrate consumption may decrease the serum BCAA concentration, probably due to its oxidation in the skeletal muscle. Reduced concentrations of these metabolites can harm the muscle recovery process associated with the structure of the muscle fiber and minimize protein intake for the transport of nutrients, primarily hydrophobic compounds such as fat-soluble vitamins, which are crucial antioxidants associated with recovery after physical activity.

Furthermore, LDL showed increased metabolic profiles and serum concentrations, indicating that this compound can be mobilized for nutrient transport functions during physical activity. Similarly, the mobilization of cholesterol to form signaling hormones for appetite control, such as leptin, adiponectin, testosterone, and estrogen, is associated with the adaptive response to physical activity.

## Figures and Tables

**Table 1:** Anthropometric and body composition data of male volleyball athletes (*n* = 13).

Variables	Mean ± SD
Height (cm)	202.00 ± 3.74
Total body mass (kg)	87.21 ± 2.86
BMI (m/kg^2^)	23.47 ± 1.71
Fat Mass (kg)	18.41 ± 3.26
Fat-free mass (kg)	68.02 ± 8.00
Fat mass (%)	21.26 ± 2.86
Fat-free mass (%)	78.78 ± 21.18

Body mass index (BMI), Standard deviation (SD). Data are presented as mean ± standard deviation.

**Table 2:** Energy and nutrient intake of male volleyball athletes (*n* = 13).

Variables	Mean ± SD	Recommendation [Reference]
Energy (kcal/day)	3752.26 ± 1388.38	
PTN (g/kg)	1.96 ± 0.89	1,2 a 2 g/kg [15]
PTN (%)	18.19 ± 4.17	
CHO (g/kg)	4.52 ± 1.14	5 a 10 g/kg [15]
CHO (%)	44.54 ± 8.21	
LIP (g/kg)	1.85 ± 0.95	
LIP (%)	37.26 ± 8.03	20 a 35% [15]
Calcium (mg)	1246.78 ± 678.70	1000 [25]
Iron (mg)	34.70 ± 15.21	8 [25]
Potassium (mg)	3897.32 ± 926.70	3.400 [25]
Zinc (mg)	24.10 ± 12.51	11 [25]
Selenium (µg)	246.38 ± 123.44	55 [25]
Sodium (mg)	4923.95 ± 2141.12	1500 [25]
Vitamin A (µg)	1682.032 ± 1918.29	900 [24]
Vitamin D (µg)	182.46 ± 195.14	15 [24]
Vitamin E (mg)	21.93 ± 22.94	15 [24]
Vitamin C (mg)	207.73 ± 122.19	90 [24]
Thiamine (mg)	3.68 ± 1.77	1.2 [24]
Riboflavin (mg)	4.91 ± 2.60	1.3 [24]
Niacin (mg)	58.96 ± 25.69	16 [24]
Ac. Pantothenic (mg)	15.05 ± 11.88	5 [24]
Pyridoxine (mg)	3.68 ± 2.51	1.3 [24]
Folate (µg) (mg)	931.79 ± 558.26	400 [24]
Cobalamin (µg)	15.40 ± 11.27	2.4 [24]

Standard deviation (SD), protein (PTN), carbohydrates (CHO), lipids (LIP), grams (g), kilogram (kg), percentage (%), kilocalories (kcal). milligram (mg), microgram (µg). Data are presented as mean ± standard deviation.

**Table 3:** Metabolites, chemical shift and mean and delta intensity in the moments before and after the men’s volleyball game (n = 13).

Metabolites	ppm	Mean Pre-Game Intensity	Mean Post-Game Intensity	∆T2-T1	*p*-Value	FDR
3-Hydroxybutyrate	1.08	1.02	0.46	−0.56	0.01	0.06
Formic Acid	8.46	1.40	3.12	+1.72	<0.01	0.06
Arginine/lysine ^a^	1.89	1.23	0.78	−0.45	0.01	0.06
Arginine/lysine ^b^	1.71	2.41	1.80	−0.61	<0.01	0.06
Formic Acid	8.43	1.57	3.28	+1.71	<0.01	0.06
Hypoxanthine	8.16	1.94	3.52	+1.57	0.01	0.06
Histidine ^a^	7.41	2.63	4.39	+1.76	0.01	0.07
Histidine ^b^	7.38	2.34	4.19	+1.85	0.01	0.07
Histidine ^c^	7.32	3.02	4.74	+1.72	0.01	0.07
Histidine ^d^	7.05	2.50	4.51	+2.01	0.01	0.06
Isoleucine ^a^	1.02	5.75	4.00	−1.74	<0.01	0.04
Isoleucine ^b^	0.93	9.61	7.01	−2.60	<0.01	0.04
LDL/VLDL	1.20	2.18	1.77	−0.41	0.04	0.15
Leucine	0.96	8.56	6.22	−2.34	<0.01	0.04
Tyrosine ^a^	7.17	3.71	5.36	−1.65	0.01	0.07
Tyrosine ^b^	6.87	3.55	5.28	−1.73	0.01	0.07
Valine ^a^	1.05	1.45	0.83	−0.62	<0.01	0.04
Valine ^b^	0.99	2.48	1.74	−0.73	<0.01	0.06

A paired *t*-test was performed, considering *p* < 0.05. FDR: false discovery rate, used for data interpretation. Metabolites are repeated since they elute at more than one peak. The different peaks were demonstrated by the letters (^a, b, c, d^).

## Data Availability

The original contributions presented in the study are included in the article/Supplementary Material; further inquiries can be directed to the corresponding author.

## Supplementary Materials

The following supporting information can be downloaded at: https://www.mdpi.com/article/10.3390/metabo14020115/s1, Table S1: Serum concentrations of biochemical markers analyzed in the study of professional volleyball athletes (*n* = 13).
