# Supplementary material for: A Decrease in Branched-Chain Amino Acids after a Competitive Male Professional Volleyball Game—A Metabolomic-Based Approach
Source: Metabolites. 2024 Feb 9;14(2):115. doi: 10.3390/metabo14020115 (PMC10890579; doi:10.3390/metabo14020115)
Supplement: Supplementary file 1 [file metabolites-14-00115-s001.zip › metabolites-2786590-supplementary.pdf]

metabolites

Article

A Decrease in Branched-Chain Amino Acids after a Competitive Male Professional Volleyball Game—A Metabolomic-Based Approach

Taillan Martins Oliveira [1], Tathiany Jéssica Ferreira [1], Paula Albuquerque Penna Franca [1], Rudson Ribeiro da Cruz [1], Mauricio Gattás Bara-Filho [2], Fábio Luiz Candido Cahuê [1], Ana Paula Valente [3] and Anna Paola Trindade Rocha Pierucci [1,*]

Supplementary Table S1. Serum concentrations of biochemical markers analyzed in the study of professional volleyball athletes (*n*=13).

Variables	Before<br>Mean ± SD	After<br>Mean ± SD	p-value
AST(U/l)	21.27 ± 14.27	21.36 ± 15.69	0.468
ALT(U/l)	16.48 ± 7.17	17.96 ± 6.10	0.071
Glucose (mg/dl)	76.62 ± 24.81	88.73 ± 26.31	0.669
CK (U/l)	339.46 ± 227.35	310.22 ± 233.26	0.426
Lactate (mmol/l)	12,685.76 ± 4920.20	11,327.83 ± 5883.91	0.355
Uric Acid	3.39 ± 1.11	3.32 ± 1.57	0.707
Triglycerides (mg/dl)	52.15 ± 26.99	41.43 ± 21.10	0.521
LDL (mg/dl)	70.72 ± 33.71	73.17 ± 23.97	0.533
HDL (mg/dl)	41.47 ± 15.07	44.70 ± 13.90	0.630
Cholesterol (mg/dl)	124.06 ± 58.46	131.74 ± 59.37	0.893
Hydroxybutyrate (mmol/l)	0.12 ± 0.10	0.1 ±0.07	0.902
CAT (mmol/l)	1.96 ± 0.29	2,12 ± 0.32	0.951

Total Antioxidant Capacity (CAT), low density lipoprotein (LDL), high density lipoprotein (HDL), standard deviation (SD), international units (IU), liter (l), milligram (mg), deciliter (dl), millimole (mmol). Data are presented as mean ± standard deviation.
